# Isolated metastatic extremity liposarcoma to the liver, an uncommon and transient finding

**DOI:** 10.1186/1477-7819-6-108

**Published:** 2008-10-09

**Authors:** Christopher A Garces, John D Reith, Stephen R Grobmyer, Steven N Hochwald

**Affiliations:** 1 Division of Surgical Oncology, Department of Surgery, University of Florida, Box 100286, Gainesville, Florida, 32610, USA; 2 Departments of Pathology, Immunology and Laboratory Medicine, University of Florida, Box 100275, Gainesville, Florida, 32610, USA

## Abstract

**Background:**

Extremity liposarcomas can metastasize to different areas of the body but have rarely been demonstrated to metastasize to the liver. Due to the unusual occurrence of isolated metastatic extremity liposarcoma to the liver, the optimal treatment of this condition is unknown.

**Case presentation:**

Less than one year after resection of a myxoid/round cell liposarcoma of the left lateral calf, a 61-year-old male presented with a CT scan showing a 2 cm low-density lesion in the right lobe of the liver. The lesion tripled in size over the next few months. An extensive evaluation revealed isolated disease to the liver. The lesion was surgically removed with a right hepatic lobectomy and the pathology was consistent with metastatic myxoid/round cell liposarcoma.

**Conclusion:**

Although extremity liposarcoma rarely metastasizes solely to the liver, the best chance at cure is with complete resection. Unfortunately, cure rates are very low in the setting of metastatic disease. As expected, the patient experienced progression of disease at sites outside of the liver 5 months after the liver resection.

## Background

Extremity soft tissue sarcomas are rare mesenchymal tumors with 3,900 new cases being diagnosed each year and are typically malignant fibrohistiocytoma, liposarcoma, or synovial sarcomas. Myxoid liposarcomas make up 38% of liposarcomas with round cell tumors (11%) and mixed lesions (8%) less commonly present [1,2].

Prognostic factors that impact on survival include histological grade and size [3]. The histological subtype of the liposarcoma and extent of round cell component is thought to be an important determinant of outcome. The incidence of metastatic disease is 29–33% for myxoid, 13% for round cell, and 40% for mixed [2,4,5].

Management of metastatic disease is a difficult problem with no clear consensus. Chemotherapy has had limited results outside of case reports [6,7]. The most common patterns of metastasis for myxoid liposarcomas are to the lung and retroperitoneum [2,6].

Hepatic metastases from a primary extremity soft-tissue sarcoma are rare [6]. There is one case report demonstrating the liver as the first site of spread from an extremity myxoid liposarcoma [7]. There is little information regarding the appropriate management of these lesions. We report an unusual case of a mixed myxoid/round cell liposarcoma of the extremity with an isolated hepatic metastasis, treated by liver resection. Although disease in the liver was determined to be the only site of disease upon initial tumor recurrence, the patient experienced progression of disease outside of the liver 5 months after the liver directed therapy.

## Case presentation

**Figure 1:**
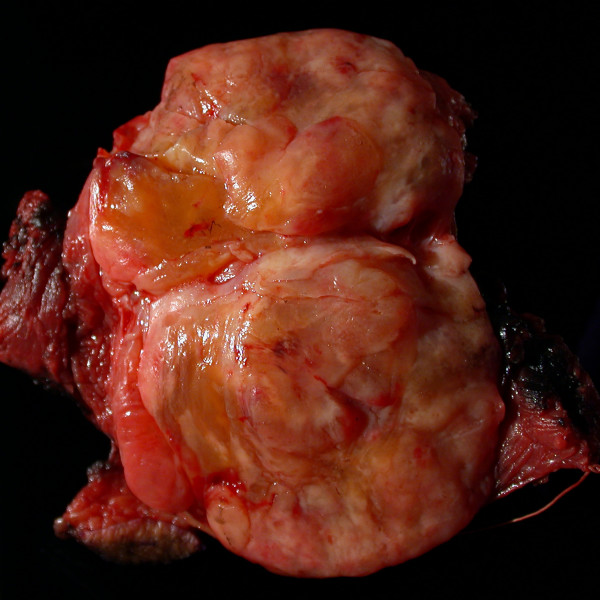
**Gross pathologic photograph of the primary tumor resected from the leg.** The tumor has a significant fat component and is relatively well circumscribed.

**Figure 2:**
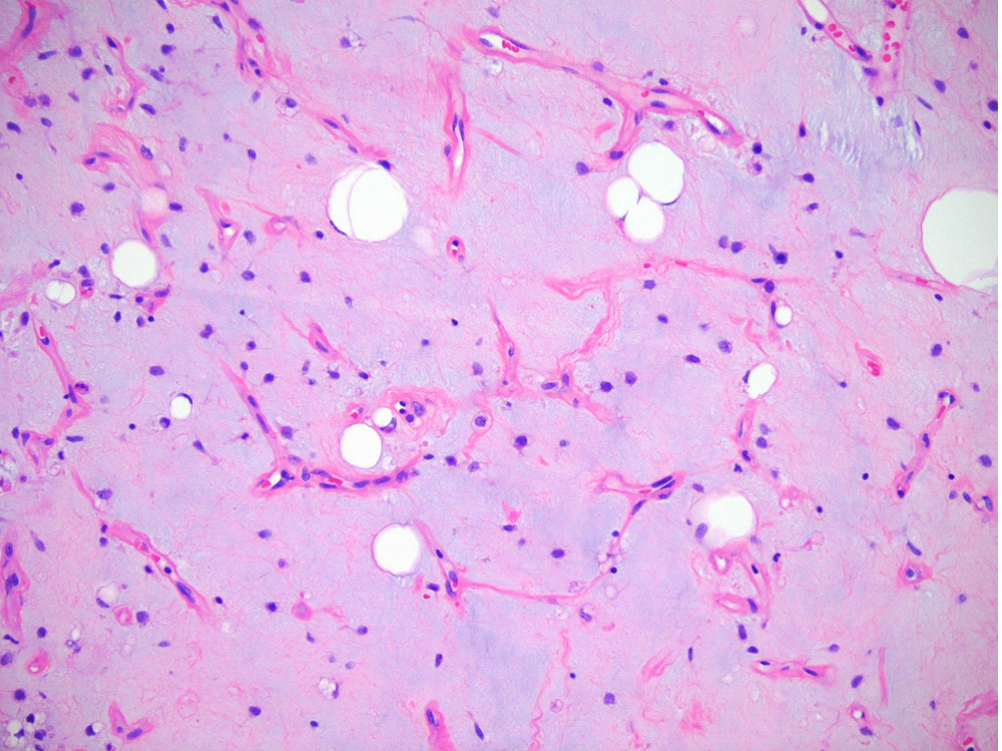
Histologic section of the primary tumor in the extremity showing a liposarcoma.

**Figure 3:**
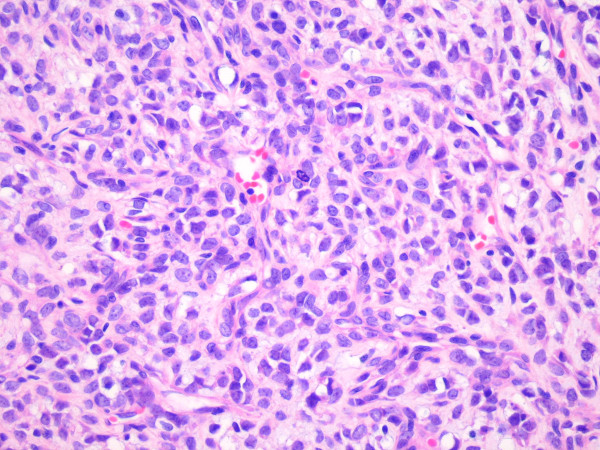
Histologic section of the primary tumor in the extremity showing a round cell component comprising < 5% of the tumor.

A 61-year-old male noted a mass in his left lateral calf. A biopsy was performed revealing a myxoid liposarcoma. The patient subsequently underwent resection of an 8.0 × 6.0 × 3.5 cm tumor with wide margins (Figure 1). Histologically, greater than 95% of the mass consisted of myxoid liposarcoma with extensive hypercellular ("transitional") foci (Figure 2), and less than 5% consisted of round cell liposarcoma (Figure 3). The tumor was classified as grade 2 of 3.

**Figure 4:**
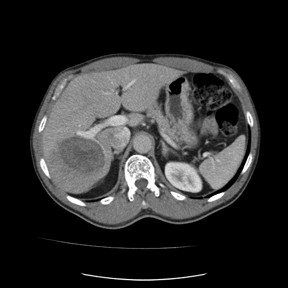
Triple-phase abdominal CT showing 7 cm low-density lesion in right lobe of liver.

The patient did well for 14 months, but follow-up computed tomography (CT) demonstrated an asymptomatic 6.3 by 6.8 cm mass in the right lobe of the liver that was growing in size (Figure 4). A PET/CT scan was done that showed the liver lesion was not FDG avid and there was no other metastatic disease. Due to concern of isolated metastatic disease to the liver, resection was recommended.

**Figure 5:**
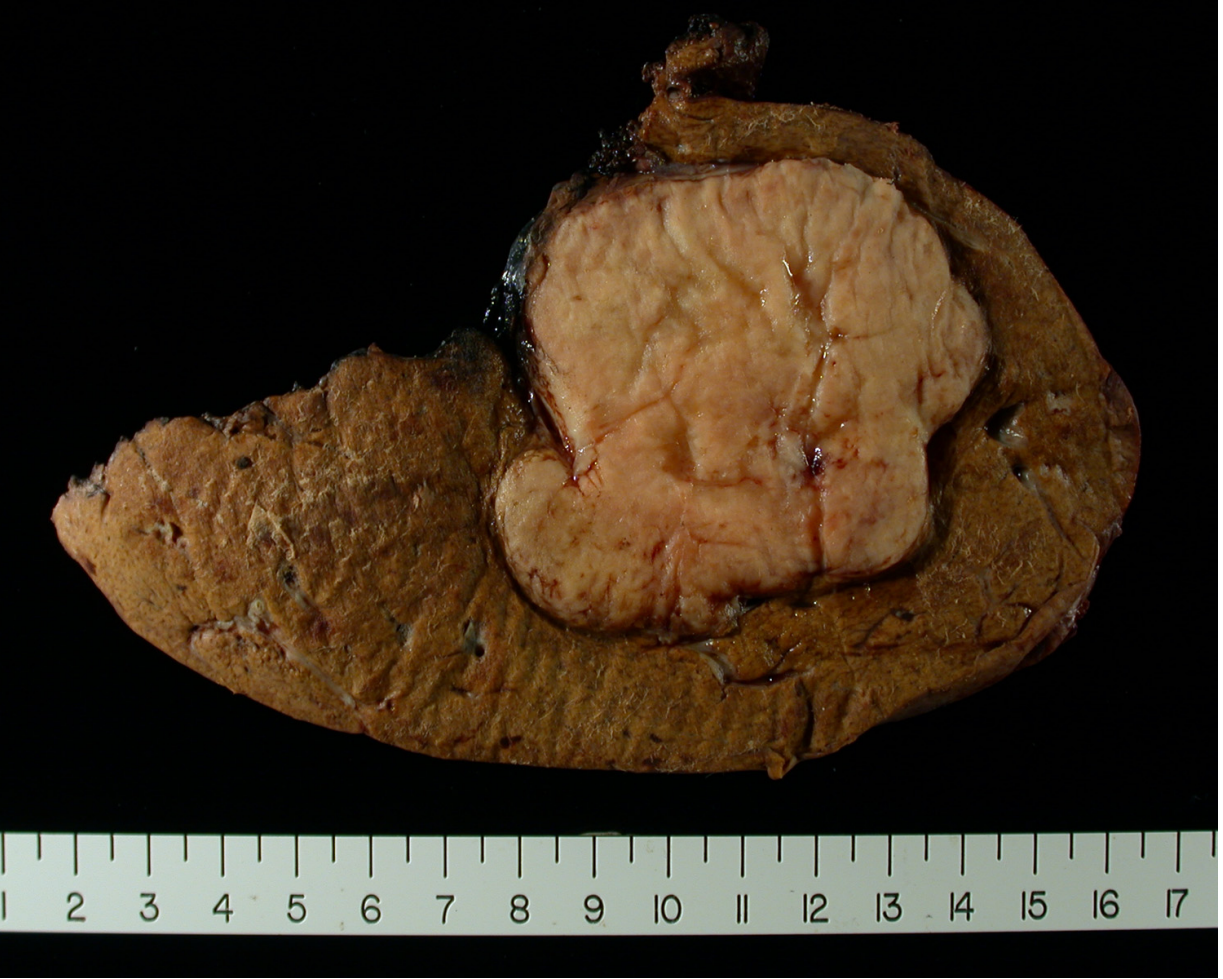
Liver resection specimen showing well circumscribed tumor with fat component.

**Figure 6:**
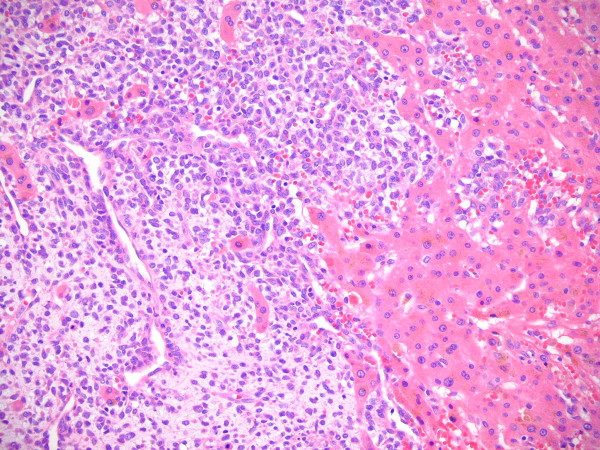
Histologic section of the metastatic disease in the liver showing extensive round cell component.

At laparotomy, ultrasound showed a large mass present in the right lobe of the liver and that the mass splayed the anterior and posterior portal pedicles apart. Next, a standard right hepatic lobectomy was performed with negative margins (Figure 5). A 7.3 × 6.5 × 7.0 cm mass of metastatic myxoid/round cell liposarcoma was present in the liver with histologic features virtually identical to those seen in the calf mass (Figure 6). The patient had an uneventful post-operative course.

Five months after the resection of the isolated liver metastases the patient was found to have a metastatic lesion on the chest wall and spinal metastases. The patient succumbed to disease during follow-up.

## Discussion

Liposarcomas consists of five subtypes: well differentiated, myxoid, round cell, pleomorphic, and mixed. The myxoid subtype is the most common variant of extremity liposarcomas and has a high predilection to extrapulmonary sites of metastasis. The most common being the retroperitoneum. A mixed myxoid/round cell component as seen in this case is found in 8% of patients presenting with an extremity liposarcoma [2]. Investigators at the Royal Marsden Hospital in London evaluated 50 patients with myxoid liposarcomas with a median follow-up of 43 months. They concluded that any round cell component of the myxoid liposarcoma was associated with a greater chance of metastatic disease [5].

The most common site of spread is to the lung from extremity sarcomas and the incidence is dependent on tumor grade and size. Hepatic metastases from primary soft tissue sarcomas (STS) frequently occur in cases of visceral and retroperitoneal tumors. It is uncommon for extremity soft-tissue sarcomas to spread to the liver (< 0.5%) [6]. It is even rarer for the tumor metastasis to be isolated to the liver, such as this case. The group at Memorial Sloan Kettering Cancer Center identified 637 patients with extremity soft tissue sarcomas and never was the liver the first or sole site for metastasis [6]. Similar results were seen at Massachusetts General Hospital. Twenty-two patients were identified with extra-pulmonary metastatic extremity myxoid liposarcoma and none of them presented with isolated liver metastases [2]. There are other case reports of extremity liposarcoma solely metastasizing to the heart, pancreas, larynx, thyroid gland, and brain [8-14]. There is only one case in the literature of an extremity myxoid liposarcoma with isolated distant metastasis to the liver [7].

There are few treatment options for metastatic soft tissue sarcomas (STS) to the liver. Conventional chemotherapy has not impacted survival in patients with metastatic STS to the liver. The group at MSKCC treated 52 of 65 patients with STS with hepatic metastases with a doxorubicin-based chemotherapy and the partial response rate was 6% with no complete responders [6]. Each patient was considered for hepatic resection, but only 14 patients were resectable. Free margins were achieved in 13/14 patients. All the patients had recurrence in the liver but median survival was 30 months in the resected group compared to 12 months in the unresected group. In this study only 4/14 resected patients had an extremity STS as their primary tumor. In addition, there were no 5-year survivors. The overall 5-year survival for pooled data of 48 patients with STS metastatic to liver who underwent hepatic resections was 11% [6].

There is a single case report of a patient with an isolated hepatic metastasis from an extremity liposarcoma who underwent resection and remained alive for 22 years [7]. This patient underwent numerous regimens for distant recurrences over the years. The regimens included doxorubicin, ifosfamide, and etoposide. The patient had stabilization of disease, but never cures of it.

Complete resection of liver disease is the only means for long-term survival in metastatic extremity liposarcoma but cure is rarely achieved. Unfortunately, the patient in this case report had a short disease free interval following liver resection and was identified to have disease on the chest wall and spine after 5 months. Consideration should be given for "adjuvant" therapy following resection of metastatic sarcoma. However, the most active chemotherapeutic options for sarcoma are of limited value and are associated with serious and potentially life-threatening toxicity. Median survival from the time metastases are recognized is on the order of 12 months, although 20% to 25% of patients with metastatic sarcoma are alive 2 years after diagnosis. In fact, the most recent study which was the largest undertaken of adjuvant chemotherapy in soft tissue sarcoma failed to demonstrate a survival benefit [15].

## Conclusion

Optimal treatment of patients with unresectable or metastatic soft tissue sarcoma requires an understanding of the natural history of the disease, close attention to the individual patient and an understanding of the benefits and limitations of the therapeutic options.

## Competing interests

The authors declare that they have no competing interests.

## Authors' contributions

CAG assembled data and participated in drafting of the manuscript. JDR assembled data and reviewed pathology. SRG participated in writing the manuscript and critical review. SNH conceived concept, assembled data, participated in drafting of the manuscript and critical review.

## Consent

Patient consent could not be obtained as the patient died, the case was presented to the Health centre Institutional Review Board of University of Florida and an IRB exemption was obtained.
